# Students’ perceptions of the instructional quality of district hospital-based training

**DOI:** 10.4102/phcfm.v8i1.1028

**Published:** 2016-07-07

**Authors:** Shehla Jabbar Memon, Jakobus Murray Louw, Martin Bac, Jannie Hugo, Waqar-un Nisa Rauf, John Edward Sandars

**Affiliations:** 1 Family Physician, Department of Family Medicine, University of Pretoria, South Africa; 2 Medical School, University of Sheffield, United Kingdom

## Abstract

**Background:**

An innovative, three-year training programme, the Bachelor of Clinical Medical Practice (BCMP), for mid-level medical healthcare workers was started in 2009 by the Department of Family Medicine, University of Pretoria.

**Aim:**

To measure the students’ perceptions of the instructional quality of district hospital-based training.

**Setting:**

Training of students took place at clinical learning centres in rural district hospitals in the Mpumalanga and Gauteng provinces.

**Methods:**

A survey using the MedEd IQ questionnaire was performed in 2010 and 2011 to measure BCMP second- and third-year students’ perceptions of instructional quality of district hospital-based training. The MedEd IQ questionnaire is composed of four subscales: preceptor activities, learning opportunities, learner involvement and the learning environment. Composite scores of instructional quality were used to present results.

**Results:**

The preceptor activities, learning opportunities and the learning environment were considered by second- and third-year BCMP students to be of consistently high instructional quality. In the area of learner involvement, instructional quality increased significantly from second to third year.

**Conclusion:**

Overall, instructional quality of district hospital-based training was high for both second- and third-year BCMP students, and the instructional quality of learner involvement being significantly higher in third year students. The MedEd IQ tool was a useful tool for measuring instructional quality and to inform programme quality improvement.

## Introduction

Healthcare workers are the cornerstone of any healthcare system, but there is a serious shortage of these workers worldwide. There is a deficit of 2.4 million doctors and nurses in 57 countries, with this shortage being particularly high in sub-Saharan Africa.^1^ In a recent report in 2015 by the International Labour Organization, the shortfall is estimated at 10.3 million healthcare workers to achieve universal health coverage.^2^ Recent reports have noted that growth in the number of healthcare professionals in South Africa has been slow and that key categories of the healthcare workforce are declining, especially in the rural communities.^3,4^

In order to improve the quality of care provided to underserved communities and address inequities in health, the World Health Organization (WHO) has called for innovative initiatives to increase the numbers of trained healthcare workers, especially in rural areas.^5,6^ The training of healthcare workers needs to be expanded through developing networks of local hospitals and primary care clinics as training sites. These environments have greater potential capacity for training than academic centres and locate training closer to underserved communities. This should enhance the recruitment of students from and the retention of professionals in those areas. It has been suggested that if healthcare professionals are recruited and trained in rural areas, they would more likely practice in rural communities.^6,7,8^

In 2009, the Department of Family Medicine at the University of Pretoria (UP) developed an innovative healthcare worker training programme leading to the Bachelor of Clinical Medical Practice (BCMP). A teaching platform in rural district and regional hospitals in the Gauteng and Mpumalanga provinces was also developed. The underpinning philosophy of learning for the new programme is that students should become self-directed learners in the class room of the real world. In this environment, the students are expected to identify their learning needs through contact with authentic clinical scenarios.^9^ Learning is facilitated by experienced family physicians and other clinicians to meet the identified learning needs. The BCMP students were allocated to, mostly rural, clinical learning centres established at district hospitals in the Mpumalanga and Gauteng provinces. The eight clinical learning centres (CLCs) are widely dispersed across the provinces and are located up to 450 km from the university campus.

The BCMP students spend a whole year at their CLC after a brief foundation course in the beginning of the year, and they also return to the university campus for a short time during the mid-year tests and end-of-year examinations.

A major concern for the BCMP programme management team was the extent to which the CLCs at district hospitals provided an adequate and satisfactory learning experience for the BCMP students. It was decided to use the MedEd IQ tool to monitor and measure student perception of the instructional quality as an indicator of the performance of the CLCs on a regular basis.^10,11^

The aim of this study was to measure the instructional quality in the CLCs at district hospitals as perceived by all second- and third year BCMP students. This study was also intended to serve as a baseline to monitor future developments in the BCMP training programme and to use the evaluation to develop interventions to improve the programme.

## Methods

### Design and sample

A structured online questionnaire was administered during the mid-year computer-based tests to all students in the second year class of 2010 and the third year class of 2011. Students from all eight CLCs at the district hospitals were included in the survey.

### Tool and data collection

The MedEd IQ questionnaire was chosen to measure student perceptions of instructional quality because previous research has noted that it is a reliable and valid instrument for assessing instructional quality in ambulatory medical settings, as well as being learner-centred.^12^

The MedEd IQ questionnaire had previously been used in the Department of Family Medicine at UP to measure the quality of education for medical students in Gauteng clinics.^13^ The MedEd IQ provides detailed and quantifiable information about the perceptions of individual students as well as of a cohort of students regarding instructional quality.

The MedEd IQ is a structured questionnaire comprising 60 statements, to be scored by respondents. This questionnaire addresses four facets (subscales) of instructional quality that contribute to learning (Appendix 1):

- Preceptor/supervisor activities (Section A of the questionnaire: 28 statements)
- Learning opportunities (Section B-1 of the questionnaire: 13 statements)
- Learner involvement (Section B-2 of the questionnaire: 9 statements)
- Learning environment (Section C of the questionnaire: 10 statements of which only the first nine were analysed because of the ambiguity of statement 10 which could be interpreted as either too fast or too slow).

### Data analysis

Analysis of the data was undertaken using STATA^®^ Version 12.1 (StataCorp). The composite scores of the students’ perceptions of instructional quality for each year were calculated for each subscale, that is, the sum of scores for items in each domain. These composite scores were the sum of the scores of all responses by each student in that year. Hotelling’s paired *T*^2^-test was used to compare the four subscales of second- and third year students’ perceptions of instructional quality presented in the observation vector (Preceptor activities; Learning opportunities; Learner involvement; Learning environment). *Post hoc* comparison with respect to individual subscales was done at the 0.0125 (0.05 divided by 4) Bonferroni adjusted level of significance; using student’s paired *t*-test.

Composite scores for each student in the subscale ‘preceptor activities’ could potentially range from 0 to 140; in ‘learning opportunities’ from 0 to 65; in ‘learner involvement’ from 0 to 27; and in ‘learning environment’ from 0 to 45.

Furthermore, the individual questions of each subscale were analysed separately, using student’s paired *t*-test, at the 0.05 level of significance.

The original scores (1–6) were on a scale of ‘strongly agreed’ (6) to ‘strongly disagreed’ (1) (reflecting the positive and negative feedback, respectively). The responses to the questions on a four-point scale (1–4) for the subscale student involvement were on a scale of ‘supervised participation with shared responsibility’ (4) to ‘no exposure’ (1).

Three statements (32, 34 and 57) were stated in the negative, and therefore, their scores were reversed.

### Ethics approval

Ethical clearance for the study was obtained from the Research Ethics Committee of University of Pretoria (approval number: 56/2011). The aim of the study was explained to the students, and written informed consent was obtained.

## Results

There was a total of 50 students who were initially accepted into the BCMP programme in 2009 and 48 were promoted to the second year. Of them, 47 completed the questionnaire in November 2010. Of the 47 students who proceeded to the 3rd year in 2011, 46 students completed the same questionnaire in November 2011. However, only 43 students completed the questionnaire correctly in both years and were therefore included in the present study, thus giving a response rate of 91%.

Ninety-five per cent of the students were of African background, and both genders were equally represented in the study group. Table 1 presents the means and standard deviations (SDs) of the composite scores of all four subscales. The results show that the students gave an average score of more than 69.5% for the lowest performing subscale in 2010 and 79% for the best performing subscale in 2011. Percentages were calculated by taking the mean as a percentage of the maximum possible score for that subscale; that is, for the lowest performing subscale in 2010, 69.5% was calculated as follows: 18.76 (mean)/27 (score range) × 100. This shows that the students rate all four aspects of their learning as positive in 2010 and 2011. The important finding depicted from this table is that there was a significant increase (*p* < 0.0001) in instructional quality (mean change of 2.60 [SD of 3.91]) in the mean composite scores of the third year group as compared to the composite scores of the second year group with respect to the subscale ‘learner involvement’. For learning opportunities an increase in instructional quality was found, but the *p*-value (0.0362) did not reach significance according to the Bonferroni adjusted level of significance (*p* < 0.0125)

**TABLE 1:** Means of the composite scores calculated for the four subscales (*n* = 43).

No	Subscale	Score range	Second year		Third year		Change in mean score (SD)	Change in % mean score	Paired *t*-test
		
			Mean score (SD)	% mean score	Mean score (SD)	% mean score			*p*-value*
1	Preceptor activities	0–140	96.84 (27.13)	69.2	96.65 (28.92)	69.0	-0.18 (32.11)	-0.2	0.9699
2	Learning opportunities	0–65	47.32 (11.39)	72.8	50.26 (10.62)	77.3	2.94 (8.87)	4.50	0.0362
3	Learner involvement	0–27	18.76 (4.25)	69.5	21.37 (4.07)	79.1	2.60 (3.91)	9.60	< 0.0001
4	Learning environment	0–45	33.23 (7.72)	73.8	32.04 (7.74)	71.2	-1.18 (7.65)	-2.6	0.3156

*Source*: MedEd IQ surveys BCMP programme, University of Pretoria

* *p* < 0.0125 of Bonferroni adjusted level of significance denotes a significant change from year 2 to year 3.

### Preceptor/supervisor activities

The test of symmetry and marginal homogeneity was applied to all of the 28 individual statements of the subscale ‘preceptor activities’. For none of these statements, a significant increase in instructional quality over two years was found.

### Learning opportunities

When the test for symmetry and marginal homogeneity was applied to the 13 individual statements of the subscale ‘learning opportunities’, it was found that there was no significant change in the instructional quality for any of the statements with regard to the learning opportunities the programme offered.

### Learner involvement

The tests for symmetry and marginal homogeneity when applied to the nine individual questions of the subscale ‘learner involvement’ showed a significant positive shift in students’ perception of instructional quality with regard to their involvement in patient care in their successive year of learning in six of the nine statements. The student’s involvement in acute, complicated and chronic patients all improved significantly and also their involvement in health maintenance, patient education and procedures. Table 2 shows the statements that changed significantly.

**TABLE 2:** Significant changes in students’ perception of their involvement in learning 2010-2011.

Statement	Mean (SD) 2^nd^ year(*n* = 43)	Mean (SD) 3^rd^ year(*n* = 43)	*p*-value*
Learner’s involvement in management of acute diseases (Q44)	3.19 (2.86)	3.42 (2.41)	< 0.0001
Learner’s involvement in management of chronic diseases (Q45)	3.30 (2.88)	3.56 (2.55)	0.0005
Learner’s involvement in health maintenance (Q46)	2.95 (2.65)	3.21 (2.20)	0.0003
Learner’s involvement in complicated cases (Q48)	2.42 (2.39)	2.86 (1.86)	0.0012
Learner’s involvement in patient education (Q50)	3.51 (2.93)	3.79 (2.79)	0.0322
Learner’s involvement in out-patient procedures (Q52)	3.42 (2.83)	3.44 (2.44)	0.0029

*Source*: MedEd IQ surveys BCMP programme, University of Pretoria

* *p* < 0.05 denotes a significant change from year 2 to year 3.

These statements indicated a positive shift in the students’ perception of instructional quality with regard to their involvement in the overall patient management.

### Learning environment

Similarly, the test of symmetry and marginal homogeneity when applied to the 10 individual statements of the subscale ‘learning environment’ showed no significant increase in perception of instructional quality for any of the statements.

## Discussion

The results of this evaluation show that the instructional quality, as perceived by the BCMP students of the two study groups (second- and third-year groups) with respect to three of the subscales of the MedEd IQ, remained stable and was satisfactory over the two years. However, their perceptions of instructional quality with regard to their involvement in the different aspects of health and patient care was *significantly* higher in the third year, indicating that students expressed an increase in perception of the instructional quality when they are given more responsibility for patient care.

In a study of first- and third-year medical students, James found that learner involvement scored the lowest for first year students and this was attributed to them being novice learners.^13^ Another similar study of final year medical students reported that they perceived good instructional quality with regard to their involvement in Gauteng clinics in 2006.^11^ Thus, the increase in perception of instructional quality in the area of learner involvement might be attributed to the progression from being a second- to being a third-year student.

In a similar study by the BCMP programme at the University of the Witwatersrand (WITS) in their first cohort of BCMP students, they found comparable results using the DREEM questionnaire.^14^ The perception of instructional quality with the learning environment among the UP students was slightly higher (73%) than the WITS students (66%) The results in both studies show that the perception of instructional quality of students in rural CLCs is higher than in the urban CLCs. In 2010 and 2011, the more rural CLCs in Mpumalanga achieved consistently higher scores than the urban CLCs in Gauteng. This trend has been consistent over the period 2010–2014 with an average score of 75% or higher for the four subscales combined. This illustrates clearly that teaching at rural district hospitals provides an appropriate setting for students to learn.

The MedEd IQ questionnaire has become an integral part of the monitoring of the expanding number of sites for teaching of the BCMP programme at UP.

We have found the use of the MedEd IQ and the student evaluation and feedback with regard to their perception of instructional quality to be invaluable in the continuous quality improvement process for the BCMP programme. The tabulated display of the students’ perception of instructional quality with respect to the composite scoring of the different subscales clearly indicated that the learning platform is stable and functioning well. In 2014, BCMP students were placed in 22 different CLCs and the bi-annual completion of the MedEd IQ questionnaire has shown how CLCs are performing over the years. Changes in the different subscores over the years clearly indicated where there was improvement or a problem in certain CLCs. In a report by Pater in 2014, it was clearly shown how the worst performing CLC in 2011 improved dramatically after an intervention to clarify possible misconceptions about the BCMP programme and subsequently scored at the same level of instructional quality as other CLCs in 2013 with a mean perception of instructional quality score of 75%.^15^ In view of the above findings, we recommend a bi-annual monitoring system with feedback from students, using the MedEd IQ tool. Previous studies have demonstrated that the longitudinal use of students’ course evaluation satisfaction questionnaires with specific feedback can help to identify strategies to improve the learning experience.^14,16^ Because the BCMP programme is a new programme which is evolving continuously, monitoring of the impact of changes made in the programme is critical to improve and strengthen the CLCs and identify aspects in the programme or CLCs that need extra attention. We recommend that feedback should be given to each CLC and that the experiences and best practices at different CLCs should also be shared during bi-annual workshops at the university and at district meetings.

The BCMP programme at UP, as part of a national programme, is one of the youngest of the 20 AMTC (accelerated medically trained clinician) training programmes in Africa, and post-2015 the integration of these AMTC workers will play an important role in meeting the health needs of rural and underserved communities in Africa and South Africa.^17^

Moreover, development of academic clinical leadership in rural medical education has been identified as one of the most important challenges in low- and middle-income countries, including South Africa.

Development of rural clinical academic leadership is complex and requires time to realize in a meaningful manner. Service and training issues are inextricably linked and the capacity-building of both must be continuous to ensure sustainability.^18^

A limitation of this study is the small sample size and that this is the first cohort of the BCMP programme at UP. Another limitation is the potential bias of self-reporting by students, but there is a debate, with some studies showing that students’ ratings can be influenced by a variety of factors, including class size, time of day and attractiveness of the instructor, but other research suggests that students’ ratings are valid and reliable.^13,19^

## Conclusion

This study shows that overall the BCMP students have high perceptions of the instructional quality at their CLCs in district hospitals. The MedEd IQ appears to provide a simple evaluation method that can provide data to facilitate systematic comparison of different teaching sites and to direct faculty development to improve instructional quality. Our findings document the evaluation of the BCMP programme at UP. The findings of this study will be of interest to other institutions that are planning to introduce the training of healthcare workers in district hospitals.

## Appendix 1

### List of MedEd IQ questions

#### Section A

Preceptor/supervisor activities

My supervisors:

2) Established an active role for me at the site
3) Prepared me for patient encounters by: Reviewing the patients history with me
4) Prepared me for patient encounters by: Prioritising pertinent issues
5) Prepared me for patient encounters by: Assigning pertinent topics to read
6) Prepared me for patient encounters by: Making follow-up appointments for patients when I was available
7) Prepared me for patient encounters by: Actually demonstrating the techniques/procedures to be performed
8) Listened well to: The patient
9) Listened well to: Me
10) Instructed me at a level consistent with my knowledge and skill
11) Brought to my attention or reinforced physical findings that I had previously not seen
12) Made sure I learned something from every patient
13) Asked questions to enhance my learning
14) Created an environment in which I felt comfortable accepting challenges, even at risk of making mistakes
15) Focused on improving my understanding: History taking
16) Focused on improving my understanding: Physical exams
17) Focused on improving my understanding: Use of laboratory tests
18) Focused on improving my understanding: Use of radiology
19) Focused on improving my understanding: Pathophysiology
20) Focused on improving my understanding: Decision-making process
21) Focused on improving my understanding: Treatment options
22) Focused on improving my understanding: The role of the healthcare team
23) Focused on improving my understanding: The importance of self-directed learning
24) Gave me specific feedback geared toward my skill improvement in: Presenting findings/cases
25) Gave me specific feedback geared toward my skill improvement in: History taking
26) Gave me specific feedback geared toward my skill improvement in: Physical examinations
27) Gave me specific feedback geared toward my skill improvement in: Assessment and clinical decision-making
28) Gave me specific feedback geared toward my skill improvement in: Documentation
29) Demonstrated the value of respecting patients preferences even when they differed from my own

#### Section B-1

Learning opportunities

The opportunities:

31) Were diverse enough to let me learn from different interesting cases
32) Were too diverse, not allowing me to develop proficiency
33) Offered me the chance to develop proficiency through repeated practice
34) Were repetitive without offering new learning opportunities
35) Increased my independence in care for patients
36) Improved my communication skills with: Other doctors
37) Improved my communication skills with: Other healthcare providers
38) Improved my communication skills with: Patients
39) Included my participation in: History taking
40) Included my participation in: Physical exams
41) Included my participation in: Assessment and clinical decision-making
42) Included my participation in: Documentation
43) Included my participation in: Patient education

#### Section B-2

Learner involvement

Involvement in the following can be best described as:

(Choose level of involvement for each: 1 = No exposure 2 = Observation only 3 = Supervised participation with little responsibility 4 = Supervised participation with shared responsibility)

44) Acute diseases
45) Chronic diseases
46) Health maintenance
47) Psychosocial problems
48) Complicated cases
49) Simple cases
50) Patient education
51) Patient follow-up
52) Outpatients procedures

#### Section C

Learning environment

54) The teaching site was well suited to involve students in patients care
55) I found that the nursing staff contributed to learning
56) I felt like an integral part of the healthcare team
57) I felt my time in the consulting room was being wasted
58) This teaching site modelled: Serving the underserved in the community
59) This teaching site modelled: Coordinating patients care among agencies, specialized and hospital
60) This teaching site modelled: Responsibility for all patients care needs
61) This teaching site modelled: Developing personal relationships with patients over time
62) This teaching site modelled: Examining the social cultural context of illness
63) In relation to effective learning, I felt that the pace of patient care was (1 = Too slow, 6 = Too fast)*

*Question 63 was not analysed for this study.

## References

[1] Frenk J, Chen L, Bhutta ZA, et al Health professionals for a new century: Transforming education to strengthen health systems in an interdependent world. Lancet. 2010;376(9756):1923–1958. http://dx.doi.org/10.1016/S0140-6736(10)61854-5/21112623 10.1016/S0140-6736(10)61854-5

[2] Scheil-Adlung X Global evidence on inequities in rural health protection New data on rural deficits in health coverage for 174 countries. Extension of Social Security series, No. 47 Geneva: Social Protection Department, International Labour Office; 2015 Accessed 15-02-2016 http://www.social-protection.org/gimi/gess/RessourcePDF.action?ressource.ressourceId=51297

[3] Couper ID, Hugo JFM Addressing the shortage of health professionals in South Africa through the development of a new cadre of health worker: The creation of Clinical Associates. Rural Remote Health. 2014;14(3):2874. 25130766

[4] Human Resources for Health, South Africa 2030 HRH strategy for the health sector: 2012/13–2016/17, Abridged version of the HRH SA Strategy for the launch of the HRH SA Strategy [document on the internet]. University of the Witwatersrand; 2011 Accessed 25-07-2015 http://www.gov.za/sites/www.gov.za/files/hrh_strategy_0.pdf

[5] World Health Organization, Global Health Workforce Alliance, Geneva Scaling up, saving lives, task force for scaling up education and training for health workers. Global Health Workforce Alliance: World Health Organization; 2008.

[6] World Health Organization, Geneva Increasing access to health workers in remote and rural areas through improved retention. Global policy recommendations: World Health Organization; 2010. 23741785

[7] Dolea C, Stormont L, Braichet J Evaluated strategies to increase attraction and retention of health workers in remote and rural areas. Bull World Health Organ. 2010;88(5):379–385. http://dx.doi.org/10.2471/BLT.09.070607 20461133 10.2471/BLT.09.070607 PMC2865654

[8] Humphreys J, Prideaux D, Beilby JJ, et al From medical school to medical practice: A national tracking system to underpin planning for a sustainable workforce in Australasia. Med J Aust. 2009;191:244–245. 19740041 10.5694/j.1326-5377.2009.tb02774.x

[9] Hugo J, Slabbert J, Louw JM, et al The Clinical Associate curriculum – The learning theory underpinning the BCMP Programme at the University of Pretoria. Afr J Health Prof Educ. 2012;4(2):128–131. http://dx.doi.org/10.7196/ajhpe.188

[10] Schipengrover J, James PA Measuring instructional quality in community-oriented medical education: Looking into the black box. Med Educ. 1999;33:846–53. http://dx.doi.org/10.1046/j.1365-2923.1999.00480.x 10583794 10.1046/j.1365-2923.1999.00480.x

[11] Van Huyssteen MIW, Blitz-Lindeque JJ The medical instructional questionnaire used to assess the quality of South African medical education. S Afr Fam Prac. 2006;48(2):15–15d. http://dx.doi.org/10.1080/20786204.2006.10873332

[12] James PA, Kreiter CD, Shipengrover JA, et al Identifying the attributes of instructional quality in ambulatory teaching sites: A validation study of the MedEd IQ. Fam Med. 2002;34(4):268–273. 12017141

[13] James PA, Shipengrover JA, Crosson J, et al Primary care education: Measuring instruction to improve quality. Acad Med. 2002;77:922 http://dx.doi.org/10.1097/00001888-200209000-00025 12228092 10.1097/00001888-200209000-00025

[14] Dreyer A, Gibbs A, Smalley S, et al Clinical Associate students’ perception of the educational environment at the University of the Witwatersrand, Johannesburg. Afr Prim Health Care Fam Med. 2015;7:1 http://dx.doi.org/10.4102/phcfm.v7i1.778 10.4102/phcfm.v7i1.778 PMC4564909 26245597

[15] Pater B The position and functioning of Clinical Associates in four rural district hospitals in South Africa [Bachelor thesis] Institute of Health Policy and Management: Erasmus University; 2014.

[16] Kane D, Williams J, Cappuccini-Ansfield G Student satisfaction surveys: The value in taking an historical perspective. Qual High Educ. 2008;14(2):135–155. http://dx.doi.org/10.1080/13538320802278347

[17] Cobb N, Meckel M, Nyoni J, et al Findings from a survey of an uncategorized cadre of clinicians in 46 countries – Increasing access to medical care with a focus on regional needs since the 17th century. World Health Popul. 2015;16:72–86.

[18] Doherty JE, Couper ID, Campbell D, et al Transforming rural health systems through clinical academic leadership: Lessons from South Africa. Rural Remote Health. 2013;13:2618. 23848954

[19] Brown MJ Student perceptions of teaching evaluations. J Instr Psychol. 2008;35(2):177–182.

